# 37-Day microgravity exposure in 16-Week female C57BL/6J mice is associated with bone loss specific to weight-bearing skeletal sites

**DOI:** 10.1371/journal.pone.0317307

**Published:** 2025-03-26

**Authors:** Rukmani Cahill, Elizabeth A. Blaber, Cassandra M. Juran, Margareth Cheng-Campbell, Joshua S. Alwood, Yasaman Shirazi-Fard, Eduardo A. C. Almeida

**Affiliations:** 1 Blue Marble Space Institute of Science, Seattle, Washington, United States of America; 2 Biomedical Engineering Department, Rensselaer Polytechnic Institute, Troy, New York, United States of America; 3 Human Factors and Behavioral Neurobiology Department, Embry-Riddle Aeronautical University, Daytona Beach, Florida, United States of America; 4 Space Biosciences Division, NASA Ames Research Center, Moffett Field, California, United States of America; The John Hopkins University School of Medicine, UNITED STATES OF AMERICA

## Abstract

Exposure to weightlessness in microgravity and elevated space radiation are associated with rapid bone loss in mammals, but questions remain about their mechanisms of action and relative importance. In this study, we tested the hypothesis that bone loss during spaceflight in Low Earth Orbit is primarily associated with site-specific microgravity unloading of weight-bearing sites in the skeleton. Microcomputed tomography and histological analyses of bones from mice space flown on ISS for 37 days in the NASA Rodent Research-1 experiment show significant site-specific cancellous and cortical bone loss occurring in the femur, but not in L2 vertebrae. The lack of bone degenerative effects in the spine in combination with same-animal paired losses in the femur suggests that space radiation levels in Low Earth Orbit or other systemic stresses are not likely to significantly contribute to the observed bone loss. Remarkably, spaceflight is also associated with accelerated progression of femoral head endochondral ossification. This suggests the microgravity environment promotes premature progression of secondary ossification during late stages of skeletal maturation at 21 weeks. Furthermore, mice housed in the NASA ISS Rodent Habitat during 1*g* ground controls maintained or gained bone relative to mice housed in standard vivarium cages that showed significant bone mass declines. These findings suggest that housing in the Rodent Habitat with greater topological enrichment from 3D wire-mesh surfaces may promote increased mechanical loading of weight-bearing bones and maintenance of bone mass. In summary, our results indicate that in female mice approaching skeletal maturity, mechanical unloading of weight-bearing sites is the major cause of bone loss in microgravity, while sites loaded predominantly by muscle activity, such as the spine, appear unaffected. Additionally, we identified early-onset of femoral head epiphyseal plate secondary ossification as a novel spaceflight skeletal unloading effect that may lead to premature long bone growth arrest in microgravity.

## Introduction

Understanding the effects of the spaceflight environment on biological systems is an important area of research because of health concerns associated with increased human spaceflight activities in Low Earth Orbit (LEO) and in future deep space exploration of the Moon and Mars. Our research focuses on the skeletal bone loss observed in mammalian rodent models in response to microgravity and space radiation, including their mechanisms of action, and their relative contributions to altered skeletal homeostasis. Rigorously investigating the effects of spaceflight on bone, however, has proven to be complex because of highly variable space experiments conditions, plus technical, logistic and other limitations on the ability to control and replicate those experiments.

**Fig 1:**
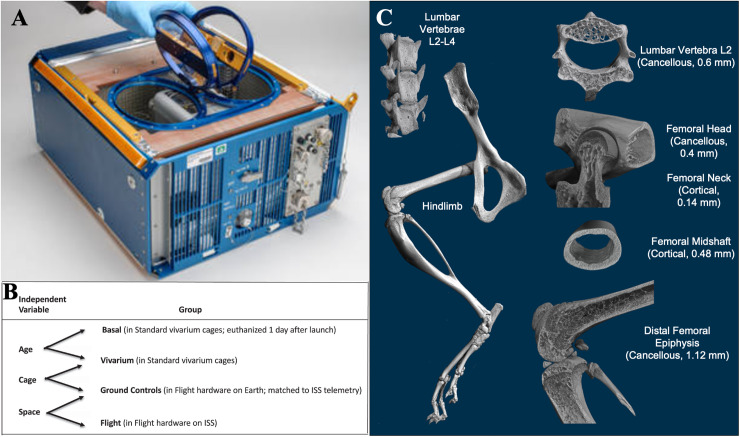
(A) Image of the RH used in the ground control and spaceflight conditions (credit Dominic Hart, NASA). (B) Each experimental condition of the NASA RR-1 study showing the biologically relevant independent variables examined. Adapted from Choi, et al. [11]. (C) Representative studied sites of μCT analysis with type of bone and the thickness of the selected VOI.

As a result, reports of spaceflight effects on bone vary; findings range from increases in mandibular bone volume to both significant and nonsignificant changes in tibial trabeculae and various cortical sites [1–8]. Similarly, meta-analyses of spaceflight bone loss across species show variable site-specificity and magnitudes [9,10] of bone structural parameters. The NASA Rodent Research validation mission (RR-1) introduced the NASA RH as an ISS National Lab facility for rodent studies in order to standardize discrepancies between spaceflight studies (Fig 1A). Importantly for experiment design, animals can also be euthanized and dissected on orbit, avoiding potential concerns about hypergravity exposure during spacecraft reentry in live animal return experiments. The RR-1 experiment included ground-based controls in the RH, baseline controls to account for effects of aging over the 37-day study, and vivarium controls to reference results to standard vivarium cage mouse housing [11,12] (Fig 1B). In our investigation of RR-1 bone tissue samples, we focused on the hindlimb and the spine to compare sites of the skeleton predominantly loaded by weight-bearing and muscle activity, respectively (Fig 1C). The experiment used mice during the late stages of skeletal maturation, that occur between 16 and 21 weeks of age, prior to onset of femoral growth plate closure and secondary endochondral ossification (SEO) [13].

A specific focus of our study is the femur because of its major weight-bearing role in the mouse. Cancellous regions in the proximal and distal femur provide structural scaffolding during cortical bone growth [14], and are normally atrophied with aging [15,16] post skeletal maturation. Similarly to aging, spaceflight induces unrecoverable resorption of trabeculae in rodents and humans [17,18] and increases fracture risk in long bones [19]. As a primary weight-bearing center in the mouse skeleton [20], the femoral head is an important study site to understand the effects of disuse on skeletal health. Opposite the femoral head, the distal epiphysis of the femur is another site of SEO and direct loading [21] by the torsional stress of keeping the hip joint and ankle aligned [22,23]. Unlike cancellous bone, the comparably low surface area to volume ratio of cortical bone makes it more resilient to bone resorption during spaceflight [18]. Finally, the femoral neck is the thinnest cortical region in this bone, and as such, it is the site of most hip fractures in elderly patients [24], making it another important analysis site for our study. For contrast to the predominantly weight-bearing femur, we also analyzed the L2 vertebra in RR-1 mice. In bipedal humans, the spine is primarily loaded by the weight of the upper body. In quadrupedal rodents, however, the L2 vertebra is thought to be loaded mainly through the articulation of the thorax and abdomen via the lumbar and abdominal muscles, rather than serving a weight-bearing role.

Thus, we hypothesize bone loss in the rodent model is a site-specific phenomenon associated with mechanical unloading of weight-bearing sites in the skeleton, rather than other systemic factors. Using standard methodology for the quantification of bone parameters with histology and microcomputed tomography (μCT) [25], within the regions of interest described in Fig 1C, we conducted global and same-animal paired comparisons of bone parameters in the femur and L2 vertebrae sites to rigorously test the idea that bone loss in LEO is specific to mechanically unloaded skeletal sites and not due to systemic effects.

## Methods

### Animals

The bone tissues used in this study were from mice in the NASA RR-1 flight experiment. 12 week old female C57BL/6J mice (Jackson Laboratories, Bar Harbor, ME) were matched by weight and assigned to groups (n =  10/group), housed in standard vivarium cages, and acclimated for 4 weeks to control experimental conditions, as previously described [11], prior to launch at 16 weeks of age. Food and water were available *ad libitum* and further husbandry details have been published [11]. All animal procedures were approved and carried out by the Institutional Animal Care and Use Committees (IACUC) at the NASA Ames Research Center (ARC) and Kennedy Space Center (KSC). Secondary dissection tissues used in this study were collected by astronauts on orbit and dissected by the RR-1 Biospecimen Sharing Program (BSP) team on the ground. Tissues were awarded by the NASA Ames Life Sciences Data Archive (ALSDA) for study to EACA (femur) and JSA (vertebrae). Reduced specimen numbers in certain sample groups, such as the femoral head, was due to sample damage that occurred during biospecimen sharing dissections.

### Rodent habitat

The RR-1 study was the first to utilize and validate NASA’s RH modules for spaceflight experiments. These were modified from previous model of AEM to support long-term housing aboard the ISS after the end of the STS program. Mice were socially housed at n = 5 per side of the RH. RHs included new long-duration exhaust filters, refillable water compartments, and were equipped with four cameras each to assess rodent health and behavior during the experiments. Details of habitat upgrades are found in Table 1 of Choi, S., et al., 2020 [11].

### Spaceflight

Mice were launched into space at 16 weeks of age and flight conditions were replicated in controls except for transport to orbit on SpaceX Dragon at twice the normal density using a RH Transporter unit on ascent. An Animal Access Unit was used on the ISS to transfer mice from the Transporter to the RH and for accessing the mice during the experiment duration. A Mouse Transfer Box transported mice from the Transporter or the RH to the Microgravity Science Glovebox for injections and on orbit euthanasia when mice were 21 weeks of age [11]. Radiation dosimetry on ISS over the duration of the experiment was measured to be about 7.4 mGy, although shielding by the RH locker and EXPRESS rack likely reduced the radiation dose compared to the rest of the ISS [11].

### Experimental protocol

RR-1 spaceflight experiments were performed as previously described in detail by Choi, et al [11]. In brief, RR-1 used 16-week-old female C57BL/6J mice exposed to microgravity for 37 days on-orbit (FL) in RHs (4 days in flight transit and 33 days on the ISS). A group of ground control mice was housed in standard mouse vivarium cages (VIV) while another group was housed in RHs (GC) (Fig 1A) inside ISS Environmental Simulator chambers (ISSES). The ground control mice were offset by 4 days relative to spaceflight to account for transit time. An additional group of basal control mice was euthanized 1 day after launch (BL). All mice carcasses were kept at -80^o^C until FL mice samples were returned to Earth (105 days). Upon thawing and dissection, femurs and a section of the lumbar vertebral column were fixed in 4% paraformaldehyde for μCT, and for paraffin embedding plus sectioning, followed by histological staining.

### Safranin-O staining

The staining of sulfated glycosaminoglycans (sGAGs) using Safranin-O was performed on 6 μm deparaffinized sections of the femoral head and distal epiphysis to obtain a representative sampling of pre-SEO and SEO samples. Cartilage was stained using 0.1% Safranin-O. Bone tissue was visualized using polarized light and differential interference contrast (DIC). In each animal, a sagittal section of each region was selected for comparisons between experimental conditions and the femoral head plus distal epiphysis, was imaged on Zeiss Axioskop microscope in DIC mode with a 20X 0.5 NA Plan NeoFluar lens (Carl Zeiss Microscopy, New York, USA) for sGAG content evaluation.

### Microcomputed tomography

The right femur from each sample was scanned using Bruker SkyScan 1272 μCT (Kontich, Belgium). Scans were performed at 60 kV of x-ray energy with a 0.25 mm thick aluminum filter. Images were taken every 0.5 degrees and averaged over 3 frames. Scans using the SkyScan 1272 were conducted at 0.6 μm for the femoral head, 3 μm for the femur, and 4 μm for vertebrae. Tomographies were reconstructed with Bruker SkyScan NRecon software and the Instarecon reconstruction server (Instarecon Inc., Urbana Illinois) using a ring artifact correction of 10, and a beam hardening correction of 40%.

### Reconstruction and segmentation

Reconstructed μCT images of the femur were uploaded to DataViewer software (Bruker SkyScan, Kontich, Belgium), and reoriented in two parts; one where the z-axis was concentrically aligned with the midshaft and distal epiphysis, and one where the z-axis was concentrically aligned with the femoral neck. Vertebrae were oriented so both lateral pedicles were parallel. Appropriate orientation was confirmed and visualized in 3D using CTVox software (Bruker SkyScan, Kontich, Belgium).

Fixed dimension volumes of interest (VOIs) were selected, and bone regions were segmented using CTAn software (Bruker SkyScan, Kontich, Belgium) for each of the five selected sites. For the L2 vertebrae, a cancellous VOI of 0.6 mm was isolated, extending from the caudal growth plate to its connection with the lateral pedicles. The femoral head was analyzed with a cancellous VOI of 0.4 mm, defined by the tissue between the end of the metaphyseal growth plate and start of the cortical femoral neck. In the femoral neck, a cortical VOI of 0.14 mm segmented the tissue connecting the end of the femoral head region to the femoral shaft. The femoral midshaft was analyzed with a cortical VOI of 0.48 mm, determined by averaging the z-position of the proximal and distal ends of the femur. Lastly, the distal femoral epiphysis had a cancellous VOI of 1.12 mm, extending from the epiphyseal growth plate towards the midshaft (Fig 1C).

### Digital image analysis

The BATch MANager program within CTAn, was used to create task lists for 2D and 3D bone analyses and executed for each of the VOIs cancellous or cortical region [25], and output measurements were obtained for comparison between experimental groups. Cancellous bone in the proximal and distal epiphyses was evaluated using percent bone volume (BV/TV, %), number of trabeculae per mm (Tb.N, 1/mm), trabecular thickness (Tb.Th, mm), trabecular separation (Tb.Sp, mm), trabecular pattern factor (Tb.Pf, 1/mm), and connectivity density (Conn.D, 1/mm^3^). Cortical bone was evaluated using percent bone area (BA/TA, %), average cortical thickness (Ct.Th, mm), endosteal perimeter (E.Pm, mm), and periosteal perimeter (P.Pm, mm).

Secondary endochondral ossification was visually evaluated in cross-sectional views of the femoral head to determining if the epiphyseal growth plate and mineralized distal cartilage had been converted to the fully ossified cancellous tissue morphology characteristic of SEO.

### Statistical analysis

We used Prism v. 10.1.0 (GraphPad Inc., Boston, Massachusetts), to conduct one-way ANOVA tests between each experimental group for each measurement to identify variability between their means, with α =  0.05 set for the threshold for significance. Tukey’s Honest Significant Difference was implemented for post-hoc analysis, and these p-values were used for significance brackets. To evaluate the magnitude of effect between housing type and spaceflight conditions, the changes of BV/TV in VIV and FL groups relative to GC was calculated. To compare percent change between groups, a one-sample t-test was implemented with a hypothetical mean of zero. A paired t-test was performed between the vertebrae, femoral head, and distal epiphysis within the same sample, which evaluated within-subject variation of bone volume at weight-bearing versus non-weight-bearing site pairs.

## Results

### Bone loss in the weight bearing femoral head and distal epiphysis cancellous bone regions depends on housing type and spaceflight in microgravity

**Fig 2:**
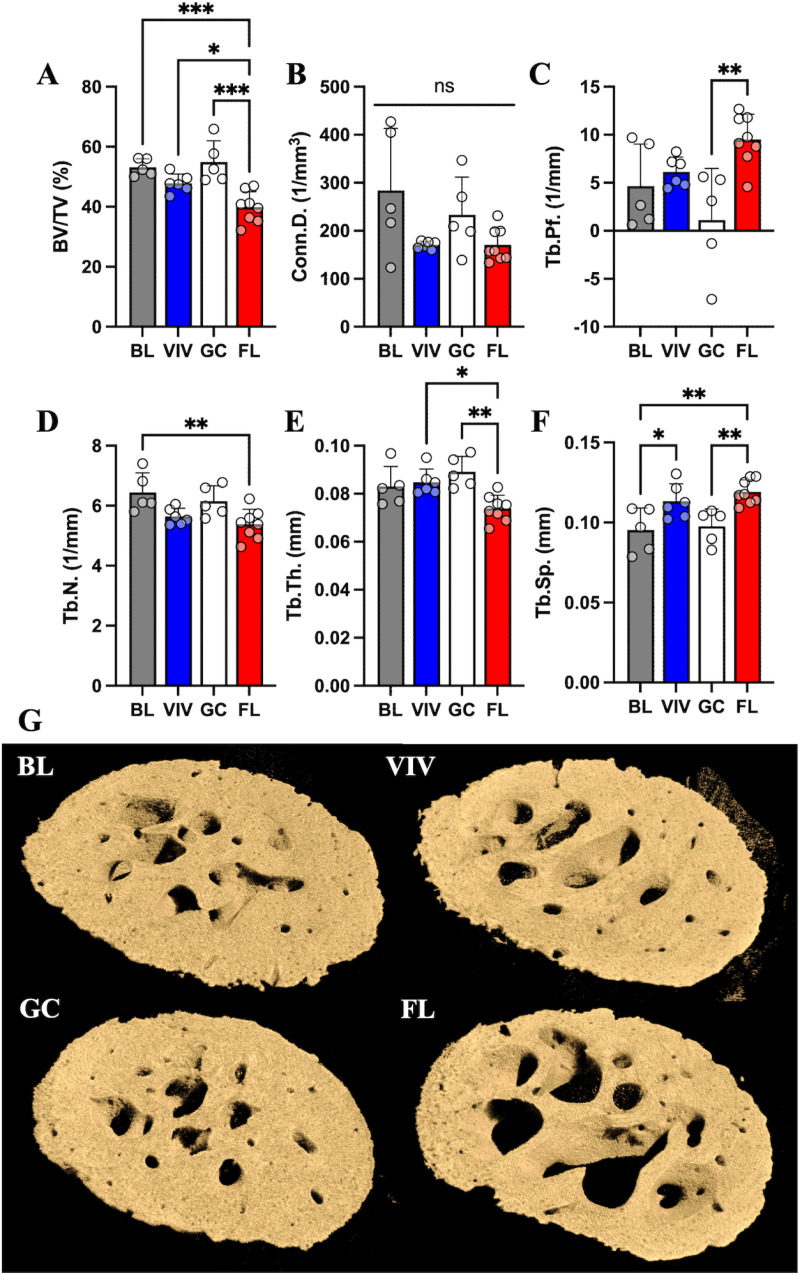
Bone loss in the mouse femoral head is associated with weightlessness in microgravity. Characterization metrics of cancellous bone in the femoral head show that (A) BV/TV is decreased in FL. Bones from FL mice have significantly lower (B) Tb.N and of those trabeculae, spaceflight reduces (C) Tb.Th. Both age and spaceflight increase (D) Tb.Sp, whereas only FL has an increase in (E) Tb.Pf. (F) Conn.D is not significantly affected; however, VIV and FL have similarly reduced Conn.D. VIV bones experience another similar increase in magnitude of Tb.Pf, which was not statistically significant. Data shown are the mean ±  standard deviation with a scatter plot (ns: non-significant, * : p <  0.033, **: p <  0.002, ***: p <  0.0002, ****: p <  0.0001). (G) μCT volumetric reconstructions of a representative sample from each group show evident decreased cancellous bone in FL.

In the femoral head, BV/TV was significantly decreased in spaceflight compared to all control environments (BL, −25%; VIV, −18%; GC, −27%) (Fig 2A). FL bones also had significantly fewer trabeculae than basal mice (−15%), which was unchanged in the two control habitats (Fig 2B). FL bones showed thinner trabeculae (BL, −16%; GC, −17%) (Fig 2C), an increase in separation (BL, +26%; GC, +22%) (Fig 2D), and a higher pattern factor (BL, +163%; GC, +755%) (Fig 2E) compared to BL and GC bones. Conn.D remained unchanged (Fig 2F). Interestingly, the VIV group had nonsignificant trends of similar magnitude in their trabecular metrics indicating loss as observed in the spaceflight group. VIV bones had more space between trabeculae than the BL control, which was not reflected in GC indicating a housing effect (+18%) (Fig 2D). Overall, the femoral head shows significant bone atrophy expressed in multiple bone parameters after 37 days in space (Fig 2G).

**Fig 3:**
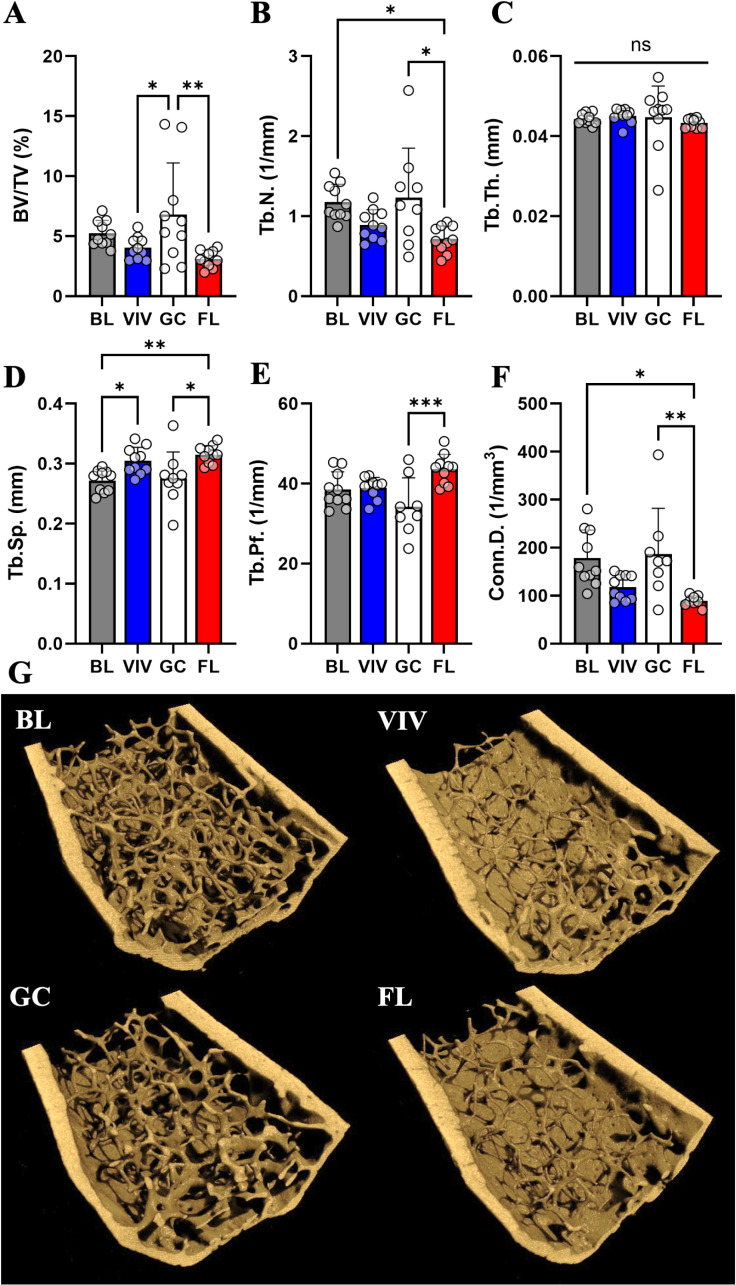
The loss of bone in the femoral distal epiphysis is affected by housing type and weightlessness conditions in microgravity. Cancellous bone measurements in the distal epiphysis exhibit a housing-related dependency on (A) BV/TV as well as an effect from spaceflight. (B) Tb.N is decreased in FL following the same patterns as BV/TV. Interestingly, (C) Tb.Th. is not affected by age, cage type, or spaceflight, however age and spaceflight significantly increase (D) Tb.Sp (E) Tb.Pf is only effected in FL. (F) Conn.D is decreased in FL. Data shown are the mean ±  standard deviation with a scatter plot (ns: non-significant, * : p <  0.033, **: p <  0.002, ***: p <  0.0002). (G) μCT volumetric reconstructions of a representative sample from each group show visibly decreased presence of bone in VIV and FL.

At the distal epiphysis, BV/TV was significantly decreased in VIV and FL bones from GC levels (VIV, −41%; FL, −55%) (Fig 3A). Similarly to the femoral head, the distal epiphysis in spaceflight samples showed fewer trabeculae as compared to BL (−39%) and GC bones (−42%), which was not observed in VIV bones (Fig 3B). Though trabecular thickness was unchanged (Fig 3C), separation of trabeculae was increased in FL compared to BL and GC (BL, +16%; GC, +14%) (Fig 3D). VIV had significantly higher trabecular separation (+12%) compared to basal mice, a result which was also observed in the femoral head. Spaceflight significantly increased Tb.Pf from GC mice (+27%) (Fig 3E). The FL group had significantly lower connectivity density than BL (−50%) and GC (−52%), which was not seen in the VIV (Fig 3F). This indicates that GC housing in the RH may rescue aging-related bone loss in the distal epiphysis of vivarium cage housed mice. In all trabecular metrics, the VIV group experienced loss trends similar to those in FL bones. Collectively, the data suggest, relative to the RH, the vivarium cage housing allows aging-related bone loss to progress in the cancellous regions of weight bearing bones in the form of fewer trabeculae and fewer plate-like structures (Fig 3G).

### Long bone cortical tissues undergo degenerative changes in space

**Fig 4:**
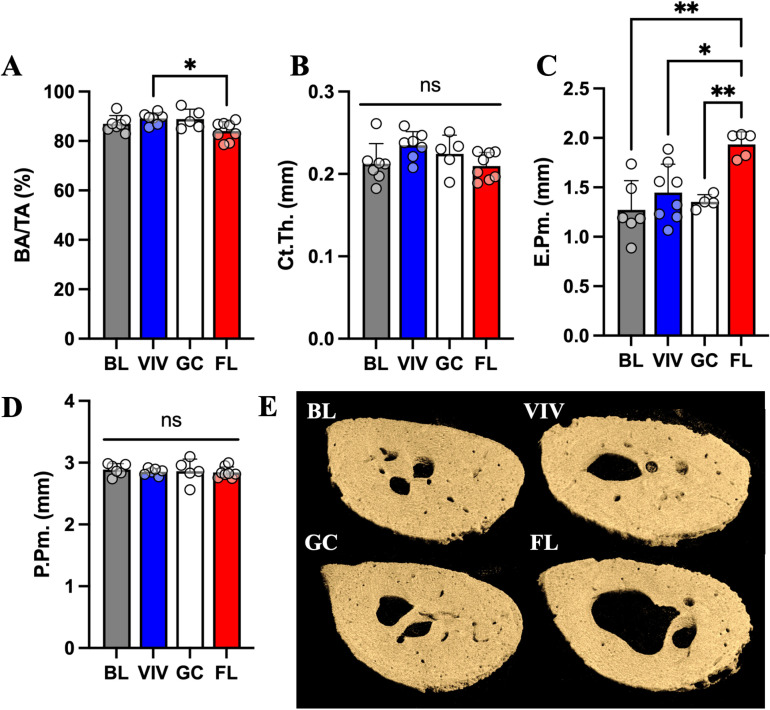
Femoral neck marrow cavity is enlarged in spaceflight but not significantly changed with age and habitat conditions. Cortical bone characterization metrics in the femoral neck indicate FL has less (A) BA/TA than VIV. Nonsignificant trends in the data show an increase in (B) Ct.Th in VIV that is opposite in FL. (C) E.Pm is significantly increased in VIV, GC, and FL from BL, though (D) P.Pm experiences no changes. Data shown are the mean ±  standard deviation with a scatter plot (ns: non-significant, * : p <  0.033, **: p <  0.002, ***: p <  0.0002). (E) μCT volumetric reconstructions of a representative sample from each group show similar presence of bone in BL and GC, while VIV and FL demonstrate erosion in the endosteal surface.

The femoral neck experiences compression and bending forces near its connection to the femoral shaft. We found that BA/TA is significantly lower in FL compared to VIV, although not by a large magnitude (−6%, p <  0.05) (Fig 4A). Ct.Th is not significantly changed between groups, though a trend exists such that the VIV group has thicker cortical bone (Fig 4B). E.Pm is significantly higher in FL than in all other groups (BL, +50%; VIV, +34%; GC, +43%) (Fig 4C), where P.Pm remains unchanged (Fig 4D). These results indicate that cancellous structures in the femoral neck endocortical region may be eroded with spaceflight (Fig 4E).

**Fig 5:**
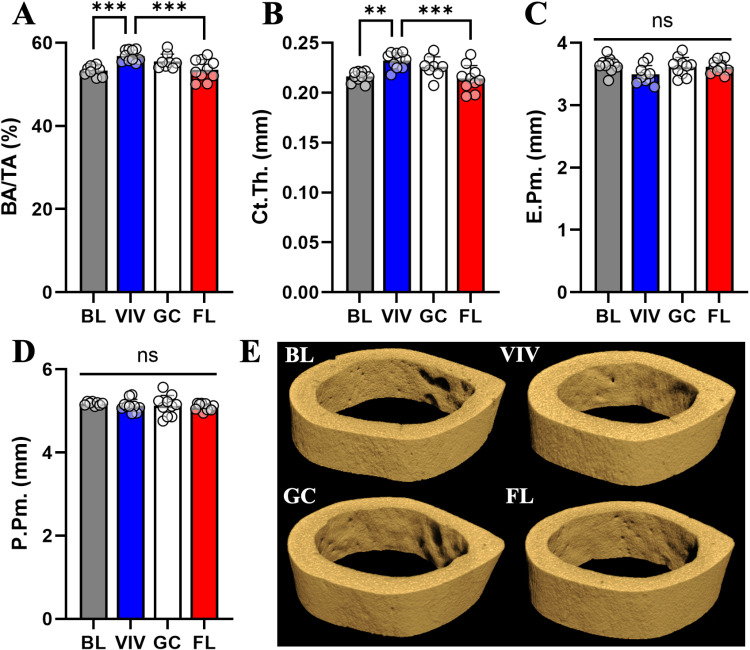
In the femoral midshaft, spaceflight abrogates increases in bone parameters BA/TA and Ct.Th in controls. Cortical bone characterization metrics in the femoral midshaft indicate the effect of age increasing (A) BA/TA and (B) Ct.Th, which is undone in FL. (C) E.Pm and (D) P.Pm are unchanged between groups. Data shown are the mean ±  standard deviation with a scatter plot (ns: non-significant, **: p <  0.002, ***: p <  0.0002). (E) μCT volumetric reconstructions of a representative sample from each group show similar presence of bone in BL and GC, while FL demonstrates erosion in the endosteal surface.

With the process of aging to skeletal maturity, cancellous scaffolding is reduced, and the cortical shell of bones becomes thicker providing more withstanding force against loading [26]. This effect is seen between the BL and VIV groups, where BA/TA (+7%, p <  0.005) and Ct.Th (+7%, p <  0.01) increase with time (Fig 5A-B). Spaceflight significantly inhibits this by reducing BA/TA (−6%, p <  0.005) and Ct.Th (−8%, p <  0.005) from VIV levels (Fig 5A-B). Unlike in the femoral neck, there were no effects seen in the E.Pm (Fig 5C), which may be due to the midshaft region lacking cancellous bone while the femoral neck has residual cancellous structures extending from the femoral head. P.Pm remained unchanged in all conditions (Fig 5D). These results indicate that the femoral midshaft experiences significant cortical thinning, which may be attributed to spaceflight (Fig 5E).

### Load-bearing vertebrae do not show μCT parameter changes related to age, housing, or spaceflight in microgravity

**Fig 6:**
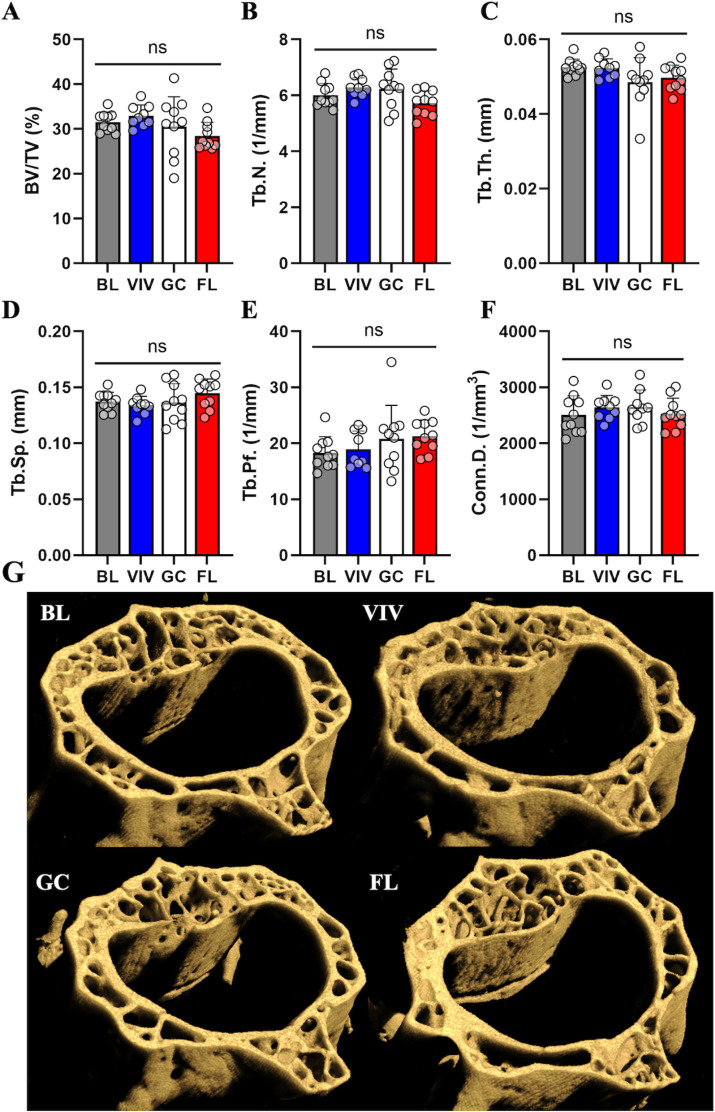
Vertebral cancellous tissueμCT parameters are not significantly affected by aging from 16 to 21 weeks, housing type, or microgravity. Cancellous bone characterization metrics in the L2 vertebrae demonstrate no differences between groups in (A) BV/TV, (B) Tb.N, (C) Tb.Th, (D) Tb.Sp, (E) Tb.Pf, or (F) Conn.D. Nonsignificant trends implicate an effect of spaceflight reducing BV/TV, increasing DA, and reducing Tb.N. Data shown are the mean ±  standard deviation with a scatter plot (ns: non-significant). (G) μCT volumetric reconstructions of a representative sample from each group show a slight decrease in bone parameters from FL mice, which are not deemed significant by statistical testing.

Within the L2 vertebrae, our analysis did not detect statistically significant changes to μCT parameters between groups, though there are several nonsignificant trends for small magnitude losses in space. Specifically, parameters like BV/TV, Tb.N, and Tb.Th in FL appear slightly decreased (Fig 6A-F), but the variance in measurements prevents any conclusions from being drawn. Thus, the L2 vertebrae is not significantly affected by housing type or spaceflight, suggesting it is not measurably responsive to weight-bearing load changes.

### Decreased mechanical loading during spaceflight in microgravity only causes significant bone loss in weight-bearing skeletal sites

**Fig 7:**
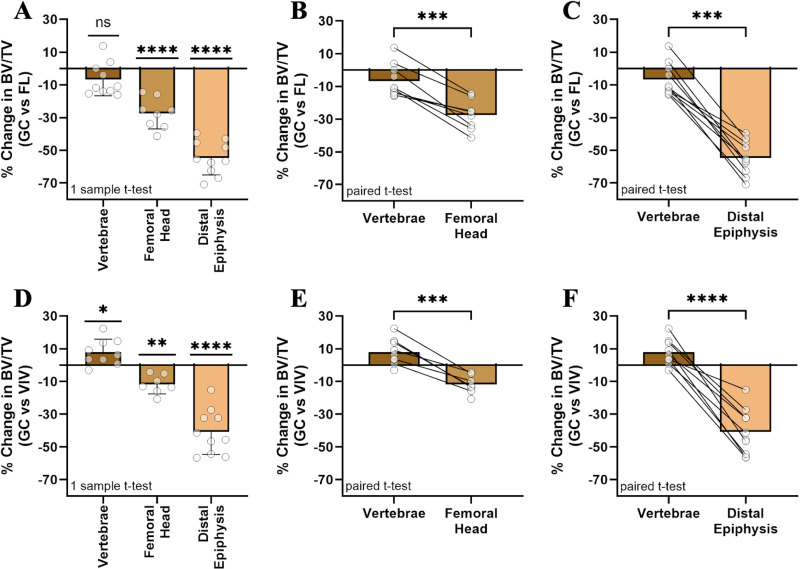
Bone loss in microgravity versus 1g controls in the RH is specific to weight-bearing skeletal sites. Using one-sample and paired t-tests, findings revealed (A) vertebrae showed no significant change, while the (B) femoral head and (C) distal epiphysis experienced significant bone loss in spaceflight, suggesting that microgravity unloading, but not other systemic factors, caused bone loss in LEO. (D-F) The vivarium cage housing, known to provide reduced topological enrichment, also elicited bone losses of smaller magnitude than spaceflight, but only in weight-bearing bone sites.

To assess if weight-bearing bone sites in the femur would experience more loss than non-weight-bearing spine, the percent change of the BV/TV of GC samples to the average value of FL was compared using a one-sample t-test with a hypothetical mean of zero (Fig 7A). The vertebrae had an average change of −7% ±  10% (p =  ns), while the femoral head lost 27% ±  9% (p <  0.0001) and the distal epiphysis lost 55% ±  10% (p <  0.0001). A paired t-test between the vertebrae and either the femoral head (Fig 7B) or distal epiphysis (Fig 7C) shows significant differences of bone loss within individual samples (p <  0.005). Throughout these results, the vivarium group in standard cages also showed bone loss effects but smaller in magnitude than spaceflight. Using a one-sample t-test and a paired t-test, these trends were quantified. The vertebrae experienced an increase in BV/TV by 8% ±  8% (p <  0.05), where the femoral head and distal epiphysis demonstrate significant bone loss of 12% ±  6% (p <  0.01) and 41% ±  14% (p <  0.0001), respectively (Fig 7D). Within samples, these effects were confirmed as all samples experienced similar degrees of loss from non-weight bearing vertebrae to the weight-bearing femoral head (p <  0.005) (Fig 7E) and distal epiphysis (p <  0.0001) (Fig 7F). Thus, the non-weight-bearing spine bone site did not experience significant change in spaceflight while weight-bearing femoral regions underwent extensive bone loss, indicating weightlessness may be the primary effector of bone loss during spaceflight in LEO.

### Secondary endochondral ossification is accelerated during spaceflight in microgravity

**Fig 8:**
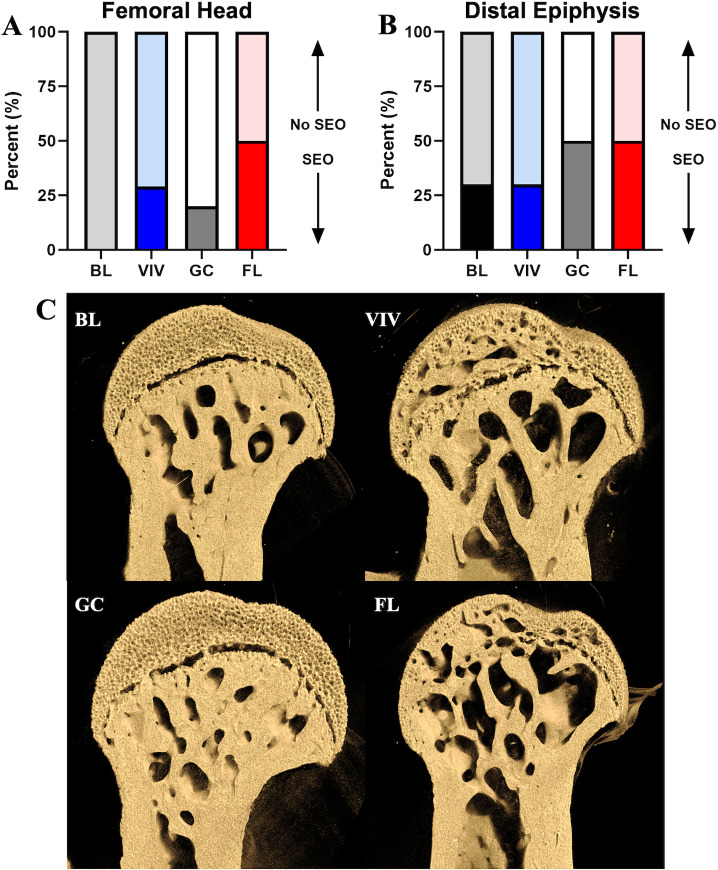
Spaceflight in microgravity accelerates secondary endochondral ossification of the proximal epiphysis in the femur. FL bones had 50% of their cohort ossified in the (A) femoral head, over twice as many as in GC cohort. Differences in ossification at BL (16 weeks) and the other groups (21 weeks) in the (B) distal epiphysis appear independent of aging. (C) μCT volumetric reconstructions of the femoral head from a representative sample from each group show increased cavitation in the mineralized cartilage of the VIV and FL groups.

Following μCT imaging, we characterized the appearance of SEO, a marker of adult skeletal maturation, at both epiphyses in the femur (Fig 8A). As SEO of the femoral head occurs between 21 and 23 weeks of age, bones from the VIV group had twice the occurrences of ossification within the femoral head than BL, and the data shows the GC habitat somewhat rescued this effect. However, FL mice had a 50% ossification rate, 20% above the VIV group, suggesting an increased prevalence of premature skeletal maturation. Interestingly, in the distal epiphysis, GC and FL, and VIV and BL groups had the same occurrences of ossification (Fig 8B), potentially related to distal epiphyseal fusion regulation predominantly by hormonal factors versus mechanotransduction [13].

**Fig 9:**
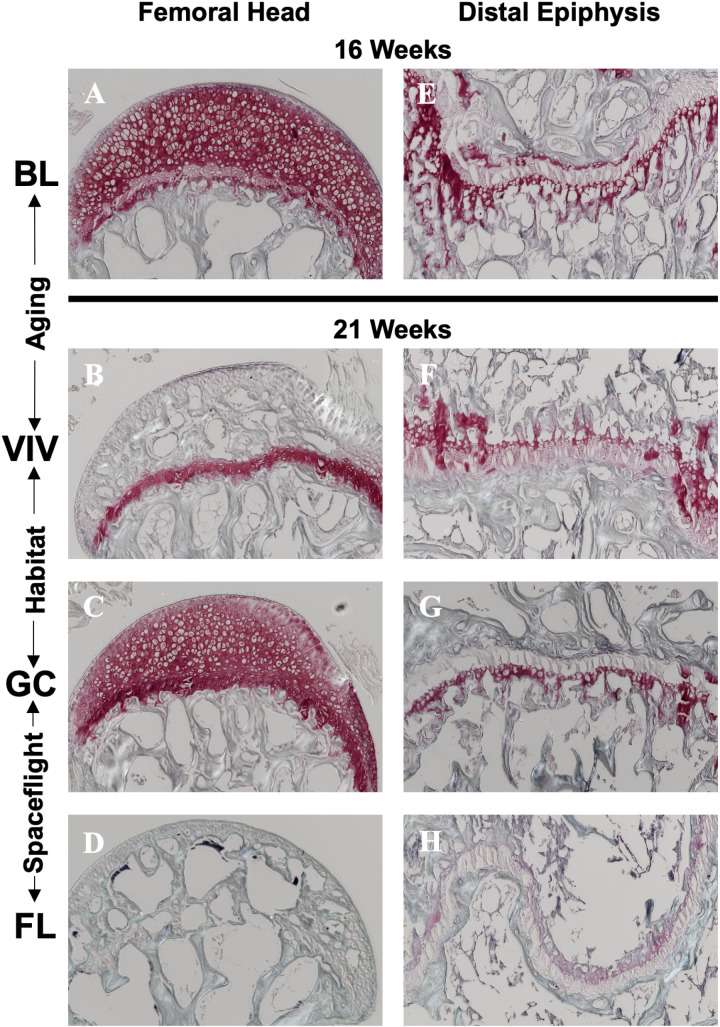
Spaceflight in microgravity reduces sGAG content in the femoral head and distal epiphysis. Representative histology of Safranin-O staining on femoral heads, with the (A) BL as the control, show a decrease of proteoglycan content in (B) VIV, a loss of the distinct tidemark in (C) GC, and complete deficit in an ossified sample from (D) FL. The distal epiphyses show ossification-dependent changes to the growth plate. During pre-ossification at 21 weeks (F, VIV), proteoglycan content decreases and is upregulated during ossification (GC, FL) to finalize remodeling into the dual-walled gap characteristic of distal epiphysis secondary ossification.

Safranin-O staining of the femoral head revealed that the region consisted entirely of hypertrophic cartilage region and a well-defined growth plate between articular cartilage and mineralized bone in baseline bones (Fig 9A). Within the VIV group, sGAG content was depleted in the articular cartilage due to early signs of ossification relative to other groups, although the growth plate remained active (Fig 9B). GC bones had this effect rescued with a maintenance of hypertrophic chondrocytes and cartilage (Fig 9C). The FL sample shows that complete ossification is associated with full sGAG and chondrocyte activity loss within the femoral head (Fig 9D). However, the distal epiphysis provides different results based on the state of SEO. From BL (Fig 9E) to VIV, both classified as pre-SEO with μCT, there is a decrease in sGAG content likely due to aging (Fig 9F). The GC sample, classified as SEO, has slightly less sGAG content than VIV, especially in the trabecular region at the top of the image (Fig 9G), however it does present a continuous area of mineralized tissue along the growth plate. This plate is marked by light pink staining, which is preserved between two continuous areas of bone in the FL example at late-stage SEO (Fig 9H). These observations suggest the process of SEO in the distal femur may induce more chondrocyte activity to rearrange cancellous structure and close the growth plate, however additional studies will be required to elucidate these questions.

## Discussion

Spaceflight in LEO is well-documented to cause significant bone loss and is often compared to physiologically aged, osteopenic phenotypes [27,28]. NASA’s early systematic studies of spaceflight effects on rodent bone physiology were conducted in short duration Space Shuttle Space Transportation System (STS) studies lasting ten to fourteen days using the Animal Enclosure Module (AEM) in a mid-deck locker. More recent results, with longer durations greater than thirty days on the International Space Station (ISS) use the Rodent Habitat hardware (RH), on the EXPRESS rack and broadly show relatively smaller degenerative bone losses possibly due to rodent acclimation to microgravity [11,19,29]. These effects are thought to be caused throughout bone tissue as a result of cellular proliferation and differentiation homeostasis imbalance between bone forming osteoblasts and bone degrading osteoclasts [30] with osteocytic osteolysis [31] and apoptosis [32] also playing a role. Exceptions to this general assertion have been reported for calvariae [33,34] and mandibles [1,35] with increases or maintenance of bone volume during spaceflight, potentially due to greater mechanical stimulation from elevated intracranial pressure due to microgravity cephalic fluid shift. However, these studies mostly measured acute, short-term spaceflight effects during NASA’s Space Shuttle (STS) era and have limitations as a model for longer-term exposures more relevant to future spaceflight missions. Other experimental variables in spaceflight include species and strain, animal age at launch, experiment duration, ambient temperature, humidity, CO_2_ concentrations, sample collection methods during or post-flight, and habitat type used. Like the STS AEM, the ISS RH provides a wire-mesh interior, allowing group-housed mice behavior and activities with associated mechanical demands similar to ground-based enriched habitats. Such activities include grappling across the cage to access food, climbing the 3D internal structure, and uniquely for space flown mice, to run in a loop along the perimeter of the habitat [12]. However, these activities only emerged after acclimation to space on days 11 and 12 in the RR-1 mice [12], where other STS experiments would have already concluded or been within a few days of ending.

A fundamental distinction between ground-based spaceflight simulations and low-Earth orbit is the type and dosage of radiation. Ground-based simulations often use low LET radiation, such as X-rays, at high acute doses of a few Gray (Gy) [36,37]. In contrast, spaceflight radiation is high linear energy transfer (LET), which has been shown to induce bone loss when acute doses of 1–2 Gy are administered [38]. However, on the ISS, the total high LET radiation dose over 37 days was only ~7.4 mGy, with an average daily dose of 207 µGy [11]. This acute dosage in ground simulations is equivalent to ~13 years of radiation exposure on the ISS delivered instantaneously. While space radiation is commonly considered a potential factor in mediating bone loss in space, the radiation measured over the duration of the RR-1 experiment in LEO is generally not considered sufficient to cause bone loss [11,39]. Therefore, conclusions drawn from ground-based simulations are not directly comparable to spaceflight conditions. Although there are other potential factors that can contribute to bone loss in space such as reactive oxygen species, calcium secretion, and hormonal imbalances [40–43], all these factors are thought to be systemic in the organism and would be expected to cause bone loss across the entire skeletal system. Current literature does not provide sufficient evidence to suggest that ROS production is site-specific within bone or bone marrow. Both microgravity and radiation are systemic factors, leading to the conclusion that ROS production affects the skeletal system uniformly. Because of this, we investigated the role of site-specific microgravity mechanical unloading in mediating bone loss, and we sought to test the hypothesis that the effects observed are primarily due to decreased weight-bearing in space and not to other systemic factors. To evaluate these ideas, we conducted μCT analysis on the femoral head, neck, midshaft, distal epiphysis, and L2 vertebrae, both from space flown mice and ground control animals housed in the topologically enriched RH as well as in standard vivarium cages.

Through aging, rodents experience decreased bone volume fraction and trabecular number due to remodeling of the bone favoring periosteal cortical development [44–46]. In contrast, dynamic mechanical loading via exercise significantly increases healthy biomechanical properties of bone [47–49]. The femoral head is also a key site of skeletal development and maturation [50], where secondary SEO of the epiphysis, converts the growth plate and mineralized cartilage into cancellous mineralized tissue [51,52]. Concomitantly, the femoral midshaft experiences high bending torsional loads during normal ambulation [53], making disuse of these areas interesting to compare to other sites of the skeleton that are not weight bearing. In the femur shaft, capacity to hold loads is primarily imparted by cortical bone which typically reaches maximum thickness after twelve weeks of age[44] with significant thinning of the cortical wall not occurring until after eight months of age [54]. Fracture risk is also increased with age [55,56] as cortical bone becomes brittle [57–61] with structural disruption [62], likely due to dysfunctional remodeling [63,64] that may prevent micro-fracture repair [65]. STS missions show insignificant acute changes in cortical bone [17], but long-term loss is resistant to post-flight recovery [66]. Thus, vertebrae might reveal potential systemic bone degenerative effects not associated with microgravity weightlessness unloading as expected in the femur. Furthermore, the RH wire mesh interior is associated with increased mouse physical activity that occurs during long-duration spaceflight after initial acclimation [12] and may load this region of the spine, possibly attenuating minor weightlessness unloading effects.

If space radiation in LEO or other systemic factors were the primary effectors of bone loss during spaceflight, we would expect systemic changes to the skeletal system. In that context, for ionizing radiation, we might also expect that thick, minerally dense regions of cortical bone could partially shield the bone marrow cavity and cancellous bone. This, however, does not appear to be the case, as our data shows the vertebral thin cortical and cancellous bone are unchanged in spaceflight (Fig 6), but the femoral neck, which has a substantial cortical region, experiences endosteal cancellous tissue loss (Fig 4). Therefore, our data strongly indicate that spaceflight-induced bone loss, is localized to two well-established direct weight-bearing skeletal locations in the femoral head and distal epiphyses. Moreover, the absence of significant bone loss in the L2 lumbar vertebra after 37 days of spaceflight is consistent with previous scans of L4 archived on NASA’s Open Science Data Repository (OSD-489, Alwood, et. al.). On Earth, the mechanical loading environment within the rodent spinal column is complex, including time- and position-varying loads of lateral bending, flexion/extension, and axial rotation dependent on the task being performed [67,68]. Despite the variation in the gravity vector with respect to the spine orientation, the vertebral body in both humans and quadrupeds experiences intermittent compressive forces and display unique fabric orientation of the cancellous tissue to the gravity vector while on Earth [68]. The response to spaceflight is less understood and likely varies by habitat topology, housing density, age, and experiment duration [10]. As active behaviors are abundantly observed in the RR-1 experiment, we infer the mice are mechanically loading their vertebral body by compression. In contrast, animals housed in the Russian Bion M-1 Habitat, with three mice per enclosure and smooth tubular walls resulting in low topological complexity [69], or in the Japanese Habitat Cage Unit, with single-housed mice and perforated walls that allowed grappling resulting in medium topological complexity [70,71], do not allow for the same running activity. To this effect, mice in the Russian Bion M-1 Habitat, significantly lost cancellous bone in the vertebral body compared to standard housing conditions [72]. Additionally, previous studies from our group using the hindlimb unloading model showed cancellous bone in the lumbar vertebra of male mice to be sensitive to musculoskeletal disuse, resulting in bone loss [73]. Taken together with the running activities and resulting intermittent compression on the ventral spine during RR-1 [12], we interpret the hindlimb unloading model to place less of a mechanical demand on the vertebral column compared to space flown and running mice.

Furthermore, we used same-animal paired t-test analyses of bone parameters in weight-bearing bones such as the femur and load-bearing vertebrae to establish, with greater statistical power than one-sample t-test or ANOVA alone, that while the former are subject to loss in space, the latter are not. Additionally, bones from mice in the RH show healthier parameters in directly loaded regions than those from the vivarium (Fig 7). Systemic effects in the vertebrae, if present, should result in weaker cancellous structures and cortical bone would not have experienced the magnitudes of endocortical erosion, as shown in our data. We acknowledge that other potential systemic changes in bone tissue [74,75] may affect mechanical properties of bone, but the data strongly suggest that increased activity and microgravity have opposite effects on bone health parameters only at specific locations within weight bearing bone and no significant effects in bones primarily loaded by muscle tension, as long as the habitat used, such as RH, is permissive of physical activity in space. The totality of the data compellingly supports mechanical unloading as the primary effector of bone loss during spaceflight in LEO.

This study also reports a previously unknown accelerated SEO effect of spaceflight on the femur. The role of SEO is to conclude the lengthening of long bones and serves as a developmental marker for skeletal maturation [51]. This process is likely mediated by transdifferentiation from chondroprogenitors to osteoprogenitors [76,77]. The results of our study suggest the femoral head initiates SEO with an increase in chondroprogenitor expression evidenced by an increase in sGAG content. Images of ossified bone demonstrate that this tidemark is propagated through the remaining articular cartilage until mineralized trabeculae constitute the entirety of the femoral head. In the distal epiphysis, the upregulation of chondrocyte activity is also demonstrated, however the plate does not dissolve to uniform mineralization, rather it maintains a layer of sGAGs between the mineralized bone in the marrow cavity and mineralized articular cartilage in the condyles. The percentage of SEO bones increased with age from BL to VIV groups. Interestingly, the effect of spaceflight on the progression of SEO was shown to accelerate complete ossification in the femoral head above the rate observed with aging. Concurrently, increased loading via activity experienced in the GC habitat mouse bones decreased the occurrence of ossification (Fig 8). Thus, bone mineralization homeostasis and SEO onset appears dependent on the magnitude of mechanical loading and unloading. Given that accelerated SEO is a significant physiological spaceflight effect, we checked additional archival μCT datasets. The 30-day Bion M-1 spaceflight experiment 19-week male mice also showed accelerated SEO, suggesting this is not an observation unique to the RR-1 study (unpublished results). This, however, was not found in other shorter flight experiments such as in the STS-131 Mouse Immunology spaceflight experiment with younger 14-week females (results in preparation). Common factors between RR-1 and Bion M-1 spaceflight experiments include a duration greater than 30 days both with mice ages nearing the onset of normal SEO which occurs at 21–23 weeks. Additional studies are needed to fully characterize the femoral head metaphyseal plate dynamics throughout the entire SEO process in the context of age, sex, and altered weight-bearing loading in microgravity.

This study supports the hypothesis that bone loss effects we observe in the 37-day RR-1 spaceflight in LEO is site-specific and likely due to mechanical unloading and not space radiation or other systemic factors. It is unlikely that low levels of space radiation, as experienced in LEO, could have systemic effects capable of mediating bone loss, as there are no reports of simulated space radiation effects at this dose and dose rate in cortical bone or lumbar vertebrae. This study, however, does not rule out the likely possibility that longer exposures, or more energetic charged particle space radiation outside of LEO will have significant bone effects. Additionally, the novel observation of accelerated epiphyseal closure in the femoral head primary weight-bearing location in mice, suggests mechanical unloading may accelerate SEO of juvenile growing long bones into a prematurely mature state. The totality of the data strongly supports mechanical unloading due to weightlessness in microgravity, not radiation or other systemic factors, as the primary effector of spaceflight-related bone loss in LEO.
